# Description of five new species of the Madagascan flagship plant genus *Ravenala* (Strelitziaceae)

**DOI:** 10.1038/s41598-021-01161-1

**Published:** 2021-11-09

**Authors:** Thomas Haevermans, Annette Hladik, Claude-Marcel Hladik, Jacqueline Razanatsoa, Agathe Haevermans, Vololoniaina Jeannoda, Patrick Blanc

**Affiliations:** 1 Institut de Systématique Évolution Biodiversité (ISYEB), Muséum National d’Histoire Naturelle, Centre National de la Recherche Scientifique, École Pratique des Hautes Études, Université des Antilles, Sorbonne Université, 45 Rue Buffon, CP 50, 75005 Paris, France; 2 Muséum National d’Histoire Naturelle, 57 rue Cuvier, 75005 Paris, France; 3 Parc Botanique et Zoologique de Tsimbazaza, Antananarivo, 101 Madagascar; 4 Département de Biologie et Écologie Végétale, Université d’Antananarivo, Faculté des Sciences, Antananarivo, 101 Madagascar; 5 Centre National de la Recherche Scientifique (CNRS), 3 Rue Michel-Ange, Paris, France

**Keywords:** Plant sciences, Natural variation in plants, Biodiversity

## Abstract

Madagascar’s emblematic traveller’s tree is a monospecific genus within Strelitziaceae, the family of the South African bird of paradise. Until now, this endemic genus consisted of a single species: *Ravenala madagascariensis* Sonn., which is grown everywhere in the tropics as an ornamental plant. The plant is immediately recognizable for its huge fan-forming banana-like leaves and is locally referred to in Magagascar by several vernacular names. “Variants” have been mentioned in the literature, but without any attempt to recognize formal taxa based on diagnostic features. In this paper, we formally describe five new species and fix the application of the name *R. madagascariensis* to the populations growing on the eastern coast of Madagascar, with the epitype growing in the marshy Fort-Dauphin area in the south. This paper has numerous implications for conservation biology and other domains of life sciences, due to the importance of this genus for the conservation of Madagascan ecosystems, the ornamental plant trade, as well as for its invasive status in several tropical areas.

## Introduction

*Ravenala* Adans.^1^, the traveller’s palm or traveller’s tree (*l’Arbre du Voyageur* in French, *ravinala* in Malagasy), is a member of the Strelitziaceae, a family within Zingiberales order whose evolutionary history is still not completely understood^2^. *Strelitzia* Banks^3^, the most species-rich genus in the family, has received considerably more attention than the other two monotypic genera *Phenakospermum* Endl.^4^ and *Ravenala*. Synflorescences of *Ravenala* are distichous and monopodial, and possess a stiff basal bract enclosing 5–20 successive bracts, each one encasing a contracted monochasium (a cincinnus); the synflorescence is itself sometimes complemented by a basal leaf-like bract. Each element of the cincinnii comprises a petal-like bracteole encasing a single flower along with the remaining of the cincinnus, each flower being perfectly hermaphroditic and not varying with their position on the inflorescence. The flower structure of *Ravenala* has been interpreted variously in the literature, Perrier^5^ describing it as similar to a *Musa* L. flower with five true segments. However, the perianth is more aptly described as consisting of three petaloid sepals and three petals^6^ (two fused sheathing the immature stamens which mechanically need to be freed to liberate the anthers, and one free petal, whose morphological characteristics are of taxonomic importance for distinguishing the various species). The flowers contain six free stamens and a style that is roughly as long as the perianth. The fruit is a dehiscent dry woody trilocular capsule, the shape of the outer parts being of taxonomic significance. *Ravenala* seeds are attached to a fatty aril which has a distinctive bright ultramarine blue color (varying from deep ultramarine blue to sky blue depending on the maturity), which contrasts with the orange aril found in the other genera of Strelitziaceae (the South American endemic *Phenakospermum* and the South African *Strelitzia*). The adaptive significance of this difference may be linked to the dispersers of the seeds, the bright orange aril being documented as an adaptation to bird dispersal, while the blue color can be an adaptation to mammal dispersal. Studies have mentioned that some lemurs see only blue and green^7–9^, or have linked the bright blue aril of *Ravenala* to the fact that perception of this color has been maintained by evolution in some Madagascan nocturnal lemurs such as aye-ayes^10^. However, Kress^11^ has also described *Ravenala* seed dispersal by birds. The morphological and ecological heterogeneity of wild *Ravenala* has been mentioned in several studies^12–15^, which were the first to consider scientifically these morphological variations. One of the differences highlighted is the seemingly variable suckering ability of *Ravenala*. This genus is usually represented as a tall suckering plant with leaves forming a perfect fan, and this can easily be appreciated from the many cultivated specimens throughout the world. However, some native Malagasy variants of the genus appear consistently devoid of suckers^13^, even when cultivated *ex situ*, while others consistently form suckers. Producing basal suckers is regarded by Tomlinson^16^ as a feature of the order Zingiberales, with the genus *Ensete* Bruce ex Horan.^17^ being the sole exception (bud neoformation can be artificially triggered in *Ensete*^18^ after removal of the main bud, but this is not true “suckering” from existing dormant buds). Solitary (non-suckering) *Ravenala* taxa are thus another exception in presenting a monocaulous monopodial architecture (Corner model^19^) within a suckering order (Tomlinson model^19^).

## Results

**Figure 1:**
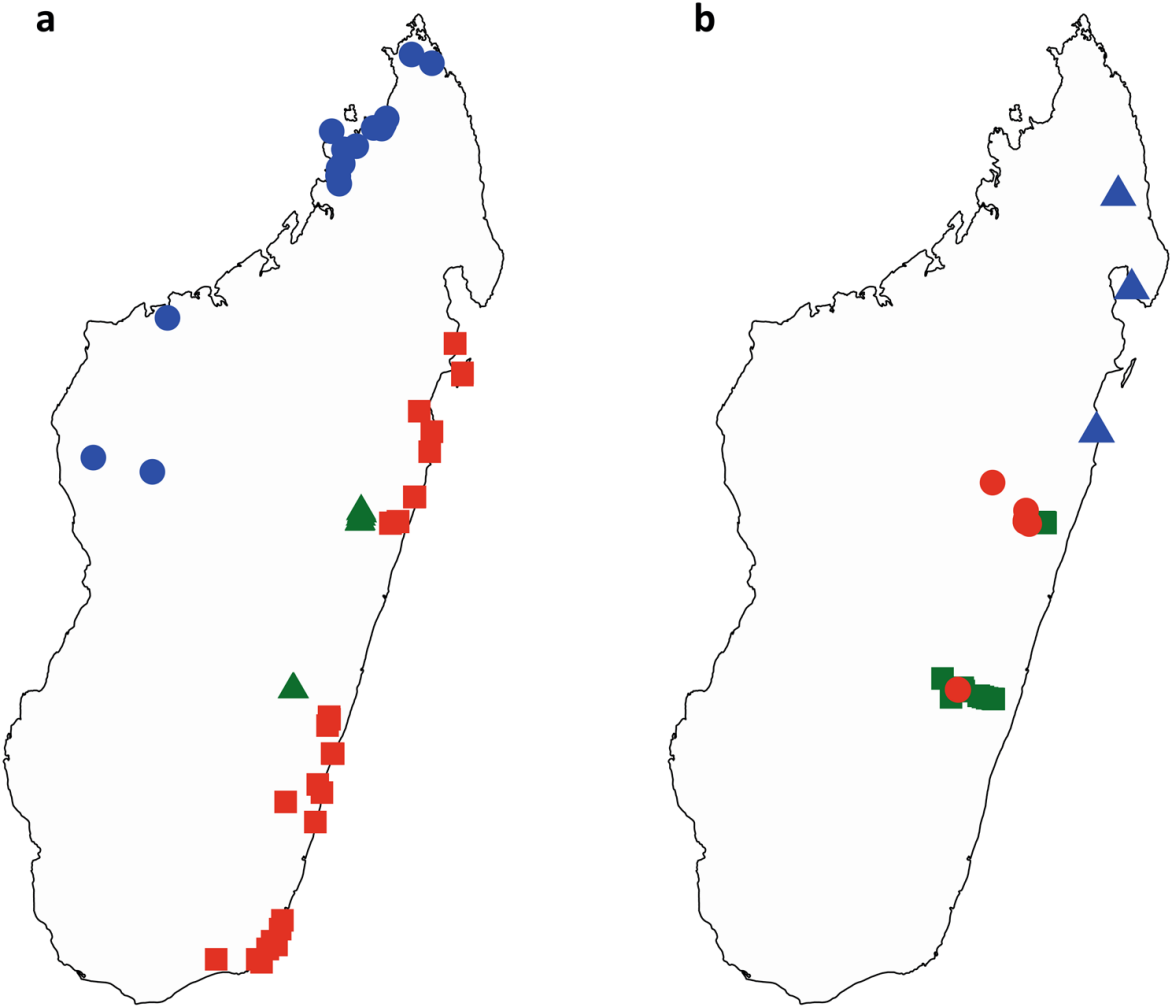
Distribution of identified specimens and observations of genus *Ravenala* in Madagascar. (**a**) *R. agatheae* (blue), *R. hladikorum* (green), and *R. madagascariensis* (red). (**b**) *R. blancii* (red), *R. grandis* (green), and *R. menahirana* (blue). Maps generated using R version 4.0.5 (https://www.r-project.org/) and Rstudio version 1.3.1093 (https://www.rstudio.com/) softwares.

**Figure 2:**
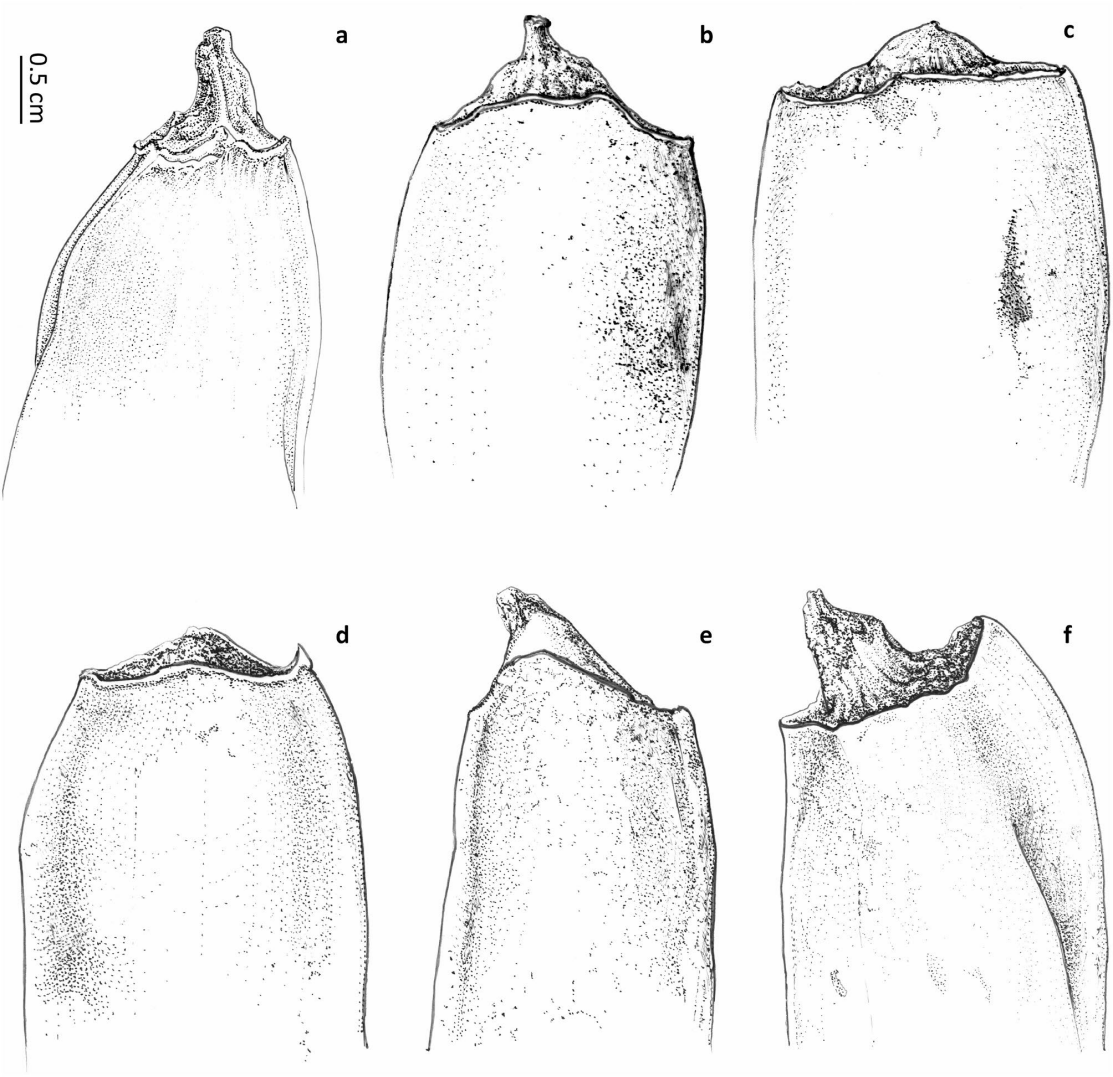
Comparison of non-opened fruit apices. (**a**) *R. agatheae*. (**b**) *R. blancii*. (**c**) *R. hladikorum*. (**d**) *R. grandis*. (**e**) *R. madagascariensis*. (**f**) *R. menahirana*. Ink drawings on $75 \, \upmu$ polyester tracing paper by Agathe Haevermans©.

Several morphotypes within *Ravenala* have been observed throughout Madagascar^12–15^, but the taxonomic study of this genus had always been impaired by the extreme difficulty of collecting these enormous plants plus the added difficulty accessing most areas in Madagascar, leading to a paucity of adequate specimens in natural history collections. Our own field and herbarium specimen observations of the various morphotypes led us to recognize and distinguish six stable units defined by observable characteristics (i.e. species), consisting of five new species in addition to *Ravenala madagascariensis*. Thus, the total number of species recognized in *Ravenala* is now similar to the number of species in its sister genus *Strelitzia*^2^ from southern Africa. Although the large *Strelitzia* species are difficult to distinguish when not in flower, *Ravenala* species can be identified when not flowering as the characters we used to define our species are observable at different developmental stages (young plants, non-fertile adults, flowering and fruiting plants, distinct flowering periods). While the distribution of the genus in Madagascar and the exact location of our type specimens can be mapped (Fig. 1), the exact extent of occurrence of each species is difficult to assess due to the aforementioned lack of available specimens. For example, in the Manombo area (eastern Madagascar), two variants are listed (the single-trunked *tokam-pototra* and the suckering *maroanaka*)^20^, but no specimens are available for scientific scrutiny to allow us to assign them to one of the taxa described here. One of the solitary morphotypes called *malama* from the Andasibe area (our *R. blancii*), may correspond to the variant called locally *fontsy ala* discovered^21^ in the Forest Reserve of Ranomafana (200 km to the south of Andasibe, at the same elevation range), but again we lack specimens to attest the presence of this taxon. This suggests that this peculiar *malama* variant could be present across a large part of the eastern coast of Madagascar, at elevations ranging from 600 m to 1,100 m^12,20^. All species except *Ravenala agatheae* are distributed along the eastern coast of Madagascar (Fig. 1), and their distribution range seems to follow an elevation gradient^20^, with *R. blancii* and *R. hladikorum* being found at the highest elevations, *R. grandis* at mid-elevation, and *R. madagascariensis* and *R. menahirana* being found at sea level (of these, only *R. madagascariensis* appears to be distributed all along the coast). By contrast, *Ravenala agatheae* from the north-western part of Madagascar is not found elsewhere on the island (Fig. 1). The major traits used to distinguish these species, as detailed in the identification key and the taxonomic treatment, are: plants suckering or strictly solitary; the flowering period; traits pertaining to the petiole such as the presence of various papery appendages or color patterns; the inflorescences; the flower structure (especially the length of the free petal *vs.* the lentgh of the two fused petals); and shape and structure of the fruit apices (Fig. 2).

## Taxonomic treatment

### Generic name

***Ravenala*** Adans.^1^ (1763: 67). $\equiv$ *Urania* Schreb.^22^ (1789: 212). –*Ravenala* Scop.^23^, *nom. illeg.* (1777: 96) as “*Ravenalla* Adans”.

**Type species** *Ravenala madagascariensis* Sonn.^24^.

**Note:** Dorr & Parkinson^25^ proposed to conserve the spelling *Ravenala* Scop. (and correct Scopoli’s original orthography “*Ravenalla*”) against *Ravenala* Adans. on the basis that Adanson’s generic names (using a uninominal nomenclature for species) were invalid. Brummitt^26^ rejected this proposal and considered that Adanson’s generic names were valid^27^ and thus that there was no need to use Scopoli’s *Ravenala* (*Ravenalla*). Moreover, the exact wording in Scopoli^23^ (1777: 96) is “*Ravenalla* Adans.”, citing Adanson explicitly, but with an incorrect spelling for the generic name (the double “l”).

### Typification and emended description

***Ravenala madagascariensis*** Sonn. (1782: 2[ed. qto.]: 223, tt. 124–126).

$\equiv$ *Ravenala madagascariensis* J.F.Gmel.^28^ (1791: 567). $\equiv$ *Urania madagascariensis* (Sonn.) Schreb. ex Forsyth f.^29^ (1794: 212). $\equiv$ *Heliconia ravenala* Willemet^30^ (1796: 22). $\equiv$ *Urania speciosa* Willdenow^31^ (1799: 7). $\equiv$ *Urania ravenalia* (Willemet) A.Rich.^32^ (1831: 19). –﻿*Ravenala madagascariensis* Adans.^1^ (1763: 597), *nomen invalid.*, appearing on page 597, abbreviated in the final index of Adanson’s book as “*Ravenala* madag. 67”, which can also be construed as referring to Madagascar as a locality.

**Type** *Lectotype, here designated: *The plate numbered 126, representing the typical lax mature infructescence, in Sonnerat^24^ (1782: plate 126). *Epitype, here designated:* MADAGASCAR $\bullet$ Fort-Dauphin, Forêt de Manantantely, [24°58′ 59.988″S, 46°55′0.012″E, calc. from label], 60–300 m elev., 15 September 1928, *H. Humbert 5730* (Epitype: MNHN-P-P02234599!, Isoepitypes: MNHN-P-P02234602!, MNHN-P-P02234604!, MNHN-P-P02234605!).

*Additional specimen examined:* MADAGASCAR $\bullet$ Toamasina: Foulpointe, Analalava Forest, plant growing close to the main forest station, 17°42.3′S, 49°27.38′E, 50 m elev., 20 March 2016, *T.Haevermans, M. Vorontsova, S. Dransfield & J. Razanatsoa 821* (TAN!, P!, K !) $\bullet$ *X. Aubriot et al. 45* (P00696168!, P00696167!, P00685124!, TAN!) $\bullet$ Along Route #5 from Fenerive to Maroantsetra, disturbed areas along road, 100 m elev., 28 February 1975, *T. B. Croat 32540* (L-WAG.1111446!, L-WAG.1111447!, MO-358490!, MO-358491!, MO-358523!) $\bullet$ Toalagnaro, Ebakika, District de Fort-Dauphin, 12 July 1932, *R. Decary 10107* (P02234596!) $\bullet$ Vondrozo (commune de Farafangana), 16 September 1926, *R. Decary 5428* (P02234588!, P02234591!, P02234592!) $\bullet$ 2 km E of Ranomafana towards Brickaville, 18.965° S, 48.8564° E, 4 March 1992, *J. Kress et al. 92-3412* (US00424302!, US00424299!, US00424300!, US00424301!, US00424303!) $\bullet$ 18 km E of Ranomafana, 25 km W of Brickaville, 18.9453° S, 48.9664° E, 4 March 1992, *J. Kress et al. 92-3414* (US00424312!, US00424309!, US00424310!, US00424311!, US00424313!). MAURITIUS $\bullet$ Isle de France, s.dat., *Commerson s.n.* (P02234587!, P-JU!, P-LAM!).

**Figure 3:**
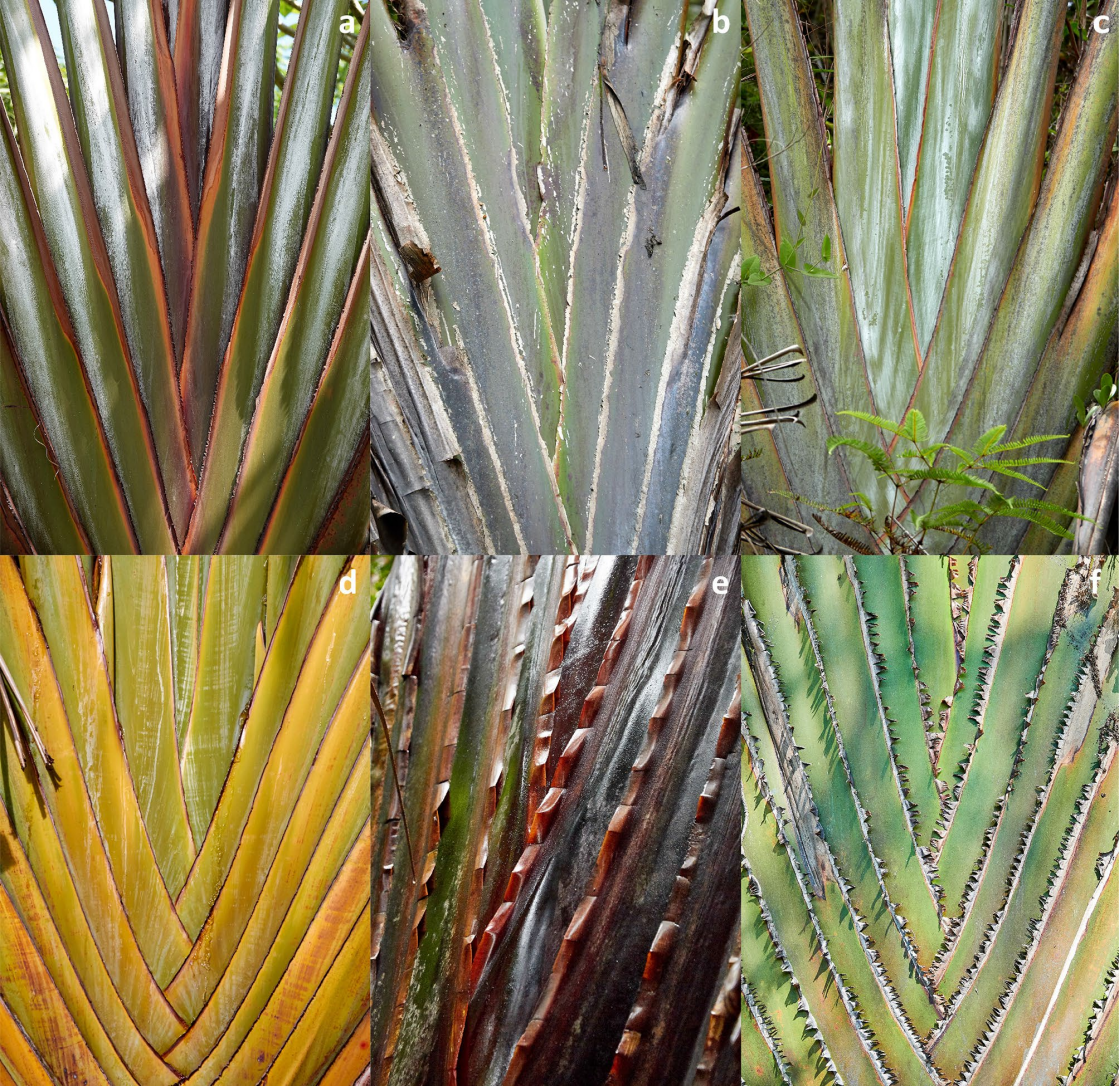
Comparison of petiole bases. (**a**) *R. agatheae*. (**b**) *R. blancii*. (**c**) *R. grandis*. (**d**) *R. madagascariensis*. (**e**) *R. menahirana*. (**f**) *R. hladikorum*. Photographs Thomas Haevermans©.

**Figure 4:**
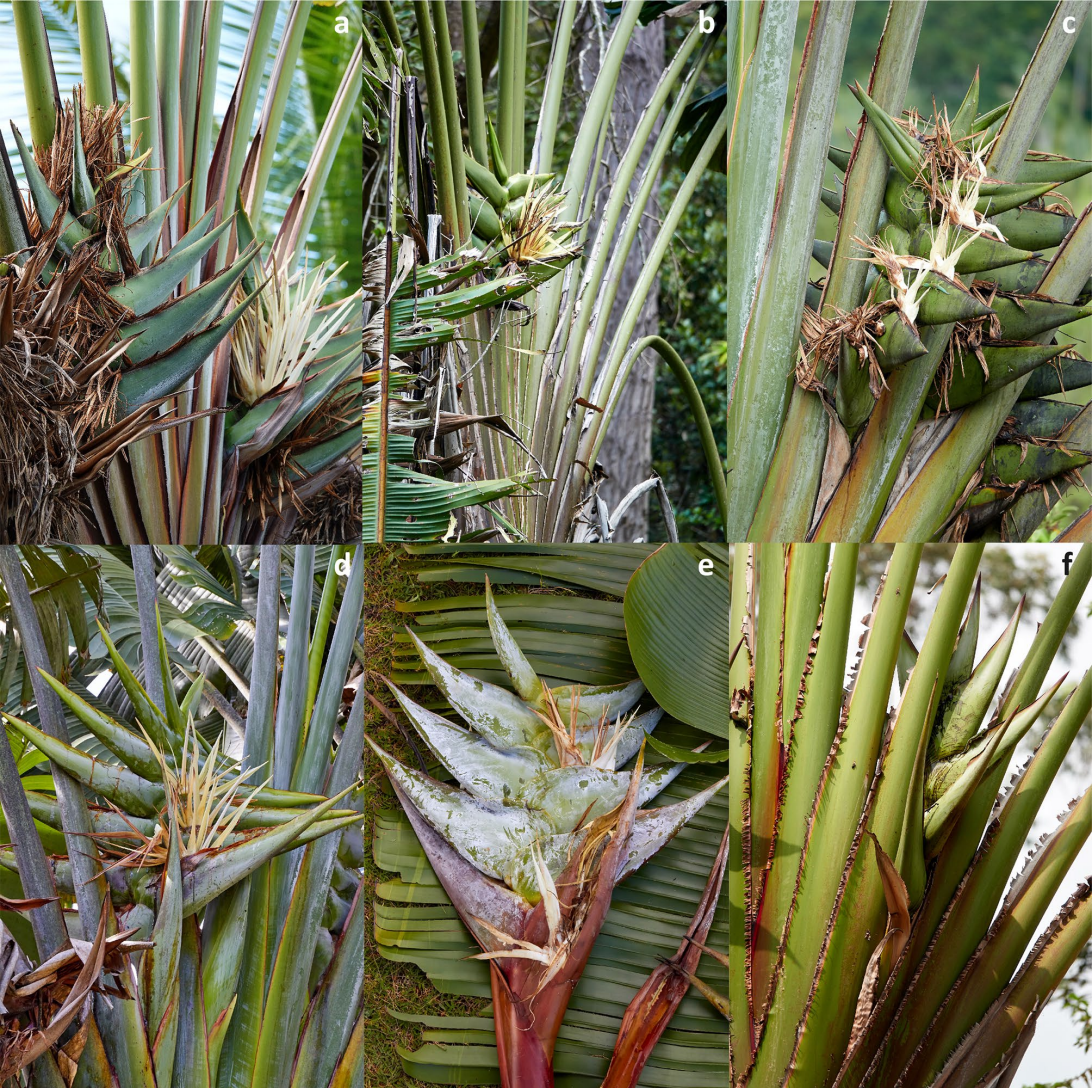
Comparison of inflorescences. (**a**) *R. agatheae*. (**b**) *R. blancii*. (**c**) *R. grandis*. (**d**) *R. madagascariensis*. (**e**) *R. menahirana*. (**f**) *R. hladikorum*. Photographs Thomas Haevermans©.

**Figure 5:**
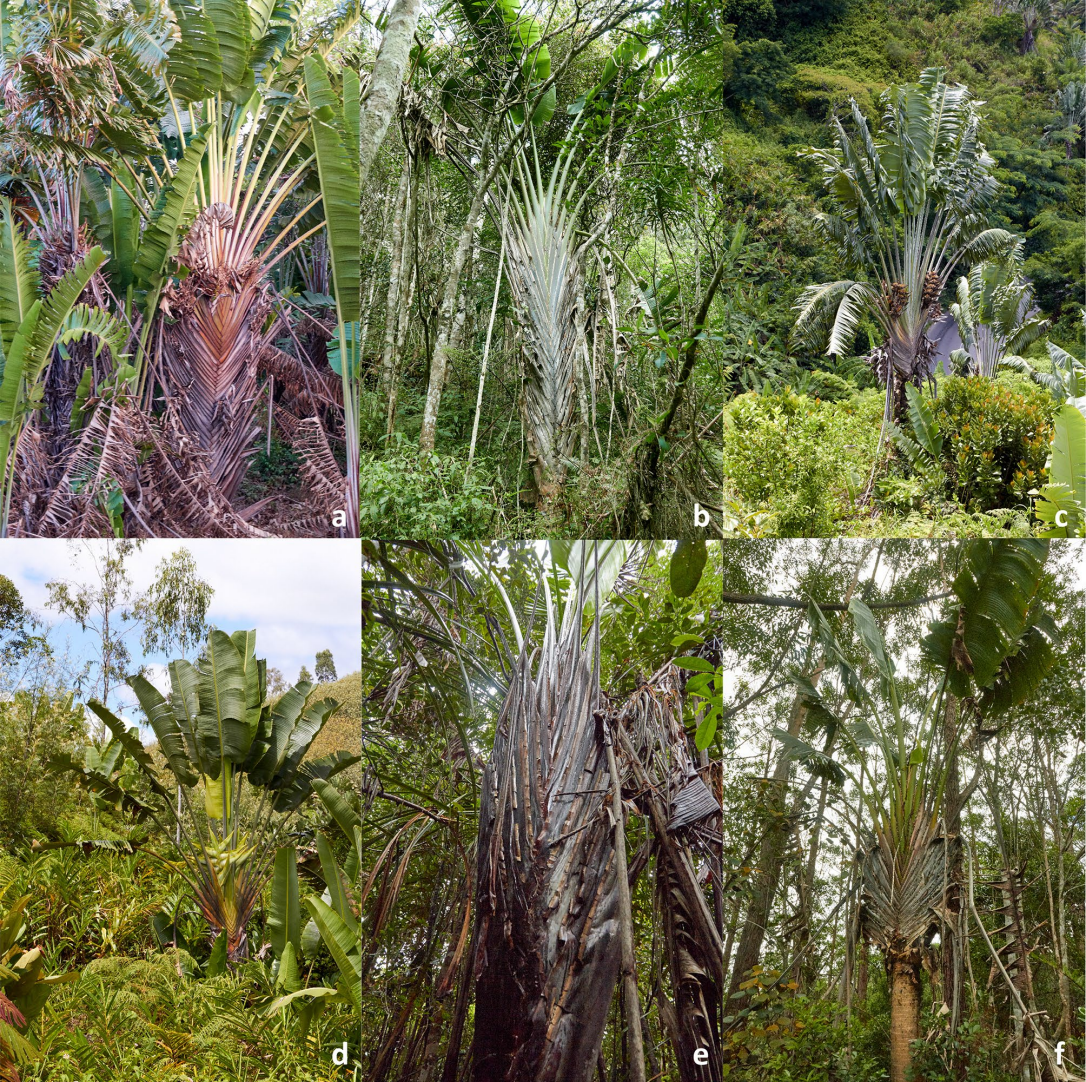
Species of *Ravenala* in their natural habitat. (**a**) *R. agatheae*. (**b**) *R. blancii*. (**c**) *R. grandis*. (**d**) *R. madagascariensis*. (**e**) *R. menahirana*. (**f**) *R. hladikorum*. Photographs Thomas Haevermans©.

***Identity of*** Ravenala madagascariensis *Sonn. *—Figs. 2d, 3d, 4d, 5d— In the absence of a specimen undoubtedly collected or seen by Sonnerat (Commerson’s specimens, collected in Mauritius and preserved in both Jussieu’s and Lamarck’s herbaria at the Paris herbarium (P-JU and P-LAM), might actually be part of original material), we decided to lectotypify from plates 124, 125 and 126 of the protologue in Sonnerat’s valid publication^24^ of the species. On page 225, Sonnerat^24^ mentions that the plant originated from Madagascar but was transported and established in Mauritius (known at the time as Isle de France) at the “Jardin des Pamplemousses”. We observed plants growing in this garden as well as naturalized plants occurring in the wild in Mauritius; all the plants we saw suckered and possessed the characteristic pointed conical fruits also observed in the Fort-Dauphin population. Sonnerat also specified that the original plant grew in marshy areas, which corresponds exactly to the coastal populations that can be found on the eastern coast of Madagascar (i.e. the “Horonorona” variant of Blanc et al.^13^). Plate 126 shows the typical mature infructescence of the species, with the space between bracts increasing before releasing the seeds (unlike other species of *Ravenala*). However, the “tree” pictured on plate 124 is a non-suckering plant, which in our opinion can be explained as artistic license on the part of the illustrator, as all the plants observed in Mauritius consistently sucker, like the plants growing in the south-eastern marshy areas. We also decided to designate an epitype with a documented locality in Madagascar (the material in P-JU and P-LAM does not bear a precise indication of locality) to fix the application of the name *R. madagascariensis* to the populations occurring in the marshy areas surrounding Fort-Dauphin, where only one morphotype is known.

**Emended description** Plants suckering, 6–12 meters tall (adult), trunk circumference (d.b.h.) 20–30 cm, juvenile and adult laminae distributed in a perfect fan, 14–25 leaves simultaneously alive on the adult plant, 1–3 leaves between inflorescences. *Leaves* adult petiole 380–440 cm long, greenish-yellow, slightly waxy, sheath margin undeveloped to moderately developed (0–9 mm), entire, not drying, slightly splitting when aged (Fig. 3d), petiole/lamina ratio 1.9–(2.2)–2.3, adult lamina $200 \times 100$ cm, light green, juvenile lamina base non-decurrent. *Inflorescences* 4–6 live lateral inflorescences at a time, $100 \times 100$ cm (peduncle excluded), 8–16 bracts per inflorescence, bracts 200–$450 \times 50$–100 mm, with some wax to very waxy, margin uniformly green (Fig. 4d), cincinnii of *ca.* 10 flowers per bract, flowering sequentially, bracteoles without a colored stripe. *Flowers* 240–280 mm long (ovary included), inferior ovary 40–50 mm long, perianth yellowish, sepals narrowly triangular 240–250 $\times 10$–12 mm, sheathing (fused) petals narrowly triangular 220–230$\times$*ca.* 10 mm, free petal acicular 180–190 $\times 5$ mm, slightly smaller than the remaining perianth with mean free petal/mean fused petal length ratio = 0.8, petal blotches absent, stamens (roughly) the same size as the perianth, 200–210 mm long, style 200–230 mm long, stigma 15–20 mm long, oblong ovoid with a basal constriction. *Infructescences* lax (bract bases not imbricate at maturity), stiff and coriaceous persisting bracts, old infructescences deciduous, 4–8 fruits per bract. *Fruits* 70–120 $\times 30$–35 mm, trilocular septifragal capsule, apices conical (Fig. 2d), seeds 6–(8.5)–$11 \times 5$–(6.4)–8 mm, shiny, dark brown, mostly globose, varying in shape according to their distribution in the capsule, ultramarine blue aril.

**Ecology** *Ravenala madagascariensis* is a low-altitude species restricted to swampy areas of the eastern coast of Madagascar. Populations outside of Madagascar on nearby islands are reputedly non-indigenous^24^.

**Preliminary IUCN assessments** We propose a Least Concern status for *R. madagascariensis*, having an E.O.O $> 20,000$ km2 and an A.O.O. $> 2,000$ km2 (criterion B)^33^.

**Note** This emended description for *R. madagascariensis* was drawn up from our own observations and collections, and was made comparable point by point to the descriptions of the five new species presented below, along with a dichotomous identification key to all six species.

### New species descriptions

#### *Ravenala agatheae* Haev. & Razanats. *sp. nov.*—Figs. 2a, 3a, 4a, 5a, 6

**Type** MADAGASCAR $\bullet$ Antsiranana: Ambanja District, along R.N.6 road to Ankaramibe, 13°45′54.8″S, 48°21′27.7″E, 30 m elev., on degraded lateritic slopes, 28 October 2018, *T. Haevermans, A. Haevermans & J. Razanatsoa 830* (Holotype: TAN!, Isotypes: K!, MO!, P!).

**Figure 6:**
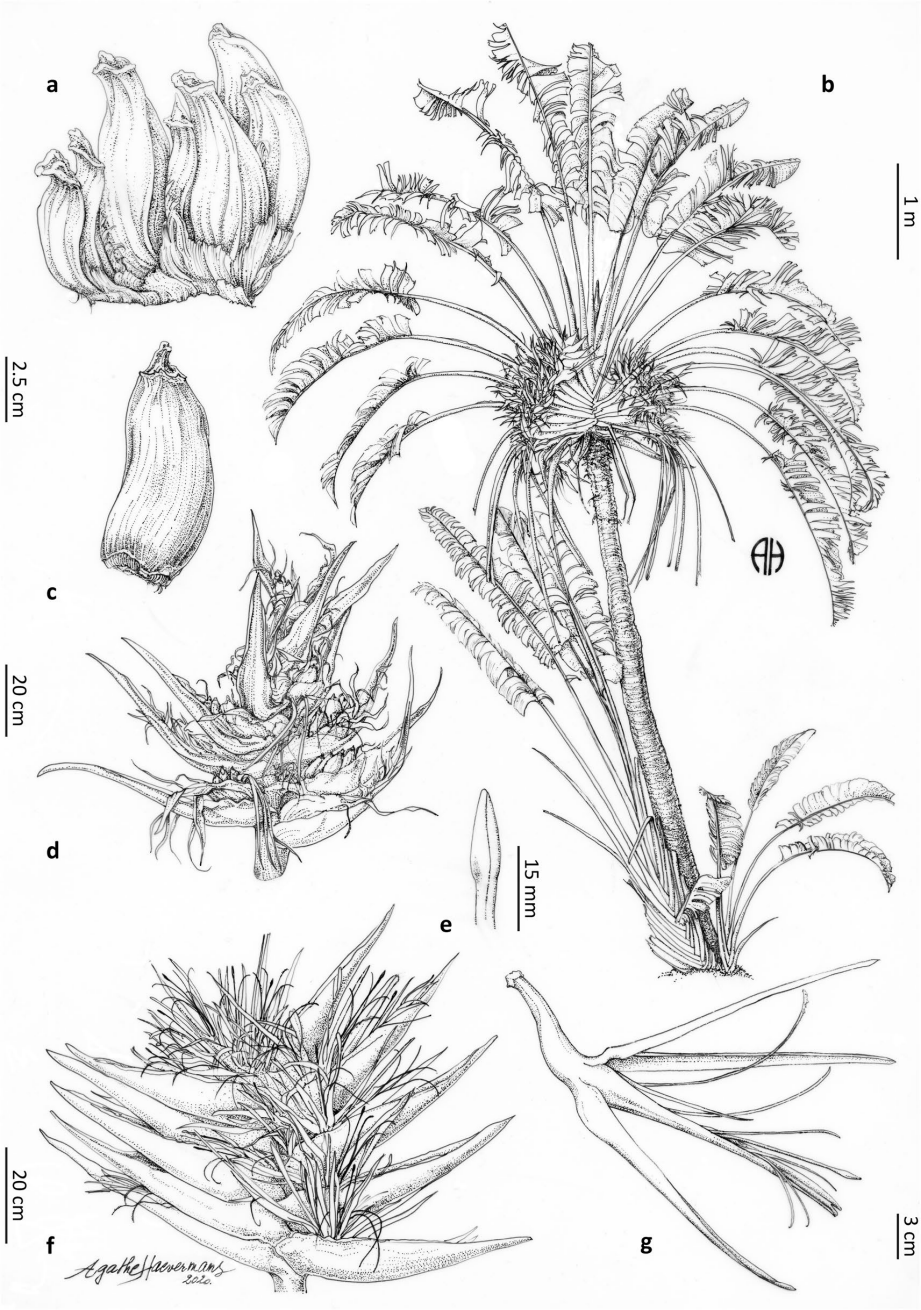
*Ravenala agatheae*. (**a**) young infructescence. (**b**) adult plant habit showing the suckers at the base and the persistent petioles and old infructescences. (**c**) fruit with a conical apex. (**d**) infructescence with remains of dried flowers and dried bracts. (**e**) style apex. (**f**) inflorescence with open flowers. (**g**) open flower. Ink drawings on $75 \, \upmu$ polyester tracing paper by Agathe Haevermans© from specimen *Haevermans et al. 830*, and observations in-situ.

**Paratypes** MADAGASCAR $\bullet$ Antsiranana: 57–58 km N of Ambanja, 13°22′59.9″S, 48°48′E, 22 May 1974, *A.H. Gentry 11878* (L-WAG.1111448!, L-WAG.1111449!, MO-358489!, TAN) $\bullet$ Ampasindava, forêts d’Ambilanivy et Rangoty, 13°48′36″S, 48°10′48″E, 29 November 2007, *L. Nusbaumer 2658* (G334213/1!, MO!, TAN) $\bullet$ Mahajanga: Morafenobe, Beravy, 15 km from Beravy, near the road from Orombato to Beravy, 18°3′50″S, 44°31′46″E, 09 June 2016, *F. Rakotonasolo et al. 2772* (K, P00782931!, TAN).

**Diagnosis** Similar to *Ravenala madagascariensis* but differs in its dark green narrower laminae, tricolor petioles with very developed dryish petiole sheath margins, very waxy petioles, the persistence of older infructescences for several years, a purple stripe on the bract margin, longer bracts, a whitish perianth, brown blotches on its mature fused petals, the bracteole apex tinged with pink, an ovoid pointed stigma, dense infructescences, smaller inflorescences, the free petal much shorter than the fused petals, and an end of year flowering period.

**Distribution** Plants restricted to Madagascar, growing in the north-western part of the island. We observed it growing from the southern part of the Diego Suarez area (on the hills along the road leading to Tsingy Rouge and the city of Sadjoavato) in the north to the western part of the Mahajanga province down to the Melaky region, with most observations around Ambanja^34^. We also observed that the species was cultivated on Nosy Be.

**Preliminary IUCN assessments** We propose a Least Concern status for *R. agatheae*, having an E.O.O $> 20,000$ km2 and an A.O.O. $> 2,000$ km2 (criterion B)^33^.

**Ecology** This species is adapted to seasonally dry and warm coastal habitats, growing on slopes at low elevations in north-western coastal areas of Madagascar, from Antsiranana (Diego-Suarez) down to the Melaky region in the Mahajanga province.

**Etymology** This species is named after to the first author’s wife, Agathe Haevermans, a botanical illustrator at the Muséum National d’Histoire Naturelle, who helped discover this species in the field with the collecting team and who contributes greatly to botany by producing illustrations of new taxa from biodiversity hotspots such as Madagascar.

**Description** Plants suckering, 6–10 meters tall (adult), trunk circumference (d.b.h.) 20–30 cm, juvenile and adult laminae distributed like a regular fan, 9–22 leaves simultaneously alive on the adult plant, 1–3 leaves between inflorescences. *Leaves* adult petiole 300–460 cm long, tricolor (dark green with a waxy white strip and red petiole sheath margin subsequently drying out, Fig. 3a), very waxy, sheath margin very developed (10 mm and more), entire, dryish-papyraceous and protruding at 90 degrees, petiole/lamina ratio 1.7–(1.95)–2.2, adult lamina 174–$210 \times 72$–86 cm, dark green, juvenile lamina base non-decurrent. *Inflorescences* 4–6 live lateral inflorescences at a time, 70–$90 \times 90$–100 cm (peduncle excluded), 10–14 bracts per inflorescence, bracts 450–500 $\times 80$– 90 mm, with some waxiness (Fig. 4a), margin bearing a purple stripe, cincinnii of 8–10 flowers per bract, flowering sequentially, some pink tinge at the apex of bracteoles. *Flowers* 260–310 mm long (ovary included), inferior ovary 40–60 mm long, perianth whitish, sepals narrowly triangular 220–250$\times$*ca.* 10 mm, sheathing (fused) petals narrowly triangular 200–$220\times$*ca.* 10 mm, free petal acicular 130–$140 \times 5$ mm, much smaller than the remaining perianth with a mean free petal / mean fused petal length ratio = 0.6, petal blotches present, stamens (roughly) the same size as the perianth, 210–220 mm long, style 220 mm long, stigma 15 mm long, ovoid-pointed with basal constriction. *Infructescences* compact (bracts bases imbricate at all stages of maturity), stiff and coriaceous persisting bracts on mature infructescence, persistence of old infructescences, 4–10 fruits per bract. *Fruits* 90–110 $\times$ 30–45 mm, trilocular septifragal capsule, apices conical (Fig. 2a), seeds shiny, dark brown, mostly globose, varying in shape according to their distribution in the capsule, ultramarine blue aril.

#### *Ravenala blancii* Haev., V. Jeannoda & A. Hladik *sp. nov. *—Figs. 2b, 3b, 4b, 5b, 7

**Type** MADAGASCAR $\bullet$ Andasibe; 18°56′00″S, 48°25′06″E; 940 m elev.; 01 December 2002; *A. Hladik & C.-M. Hladik 6760* (Holotype: TAN!, Isotypes: K!, MO!, P!).

**Paratypes** MADAGASCAR $\bullet$ Andasibe; 18°56′00″S, 48°25′06″E; 940 m elev., 23 Aug. 1998, *A. Hladik & al. 6239* (P!, fruits) $\bullet$ June 2001, *A. Hladik & al. 6650 *(P!, leaves, fruits, bracts) $\bullet$ Andasibe-Mantadia area, Vakôna, Kalonora; 18°53′17.3″S, 48°25′51.3″E, 08 November 2018, 934 m elev., *T. Haevermans & al. 832* (K!, MO!, P!, TAN!).

**Diagnosis** Similar to *Ravenala madagascariensis* but differs in its non-suckering habit, decurrent juvenile lamina bases, toroidal distribution of juvenile laminae, smaller number of leaves simultaneously alive on the adult plant, dark green lamina and green non waxy petiole, smaller leaves, smaller number of live inflorescences, smaller number of bracts in an inflorescence, non-waxy bracts, sub-simultaneous flowering, smaller flowers, smaller inflorescences, non-persistence of entire bracts on dry infructescences, October/November flowering period.

**Distribution** Andasibe-Mantadia, Ranomafana^21^. Restricted to Madagascar.

**Preliminary IUCN assessments** We propose a Data Deficient status for *R. blancii*; further fieldwork is required to understand its precise distribution and the status of its populations^33^.

**Ecology** High-elevation species found in eastern rainforests at elevations between 600 and 1,100 m. The species seems to favor cool tropical humid and shady conditions.

**Etymology** This species is named after Dr. Patrick Blanc, world renowned botanist, plant ecologist and street artist, inventor of the planted vertical walls known as “Mur Végétal” and who first recognized the sheer originality of the juvenile phases of this peculiar taxon.

**Description** Plants solitary (never suckering), 10–15 meters tall (adult), trunk circumference (d.b.h.) 20–30 cm, juvenile laminae distributed in a toroidal shape, adult laminae arranged in a regular fan, 9–16 leaves simultaneously alive on the adult plant, 2–4 leaves between inflorescences. *Leaves* adult petiole 240–310 cm long, green, not waxy, sheath margin undeveloped, entire, not drying, smooth with a worn-out irregular aspect (Fig. 3b), petiole/lamina ratio 1.8–(2.0)–2.2, adult lamina 120–160 $\times$ 90–104 cm, dark green, juvenile lamina base decurrent. *Inflorescences* 2–3 live lateral inflorescences at a time, $60 \times 70$ cm (peduncle excluded), 4–6 bracts per inflorescence, bracts 160–350 $\times$ 50–90 mm, no waxiness (Fig. 4b), margin color uniformly green, cincinnii of 5–14 flowers per bract, flowering sub-simultaneously, bracteoles sometimes pink colored. *Flowers* 165–280 mm long (ovary included), inferior ovary 40–50 mm long, perianth whitish-yellowish, sepals narrowly triangular 125–231 $\times$ 10–12 mm, sheathing (fused) petals narrowly triangular 105–190 $\times 10$ mm, free petal acicular 105–178 $\times 3$–5 mm, free petal and fused petals of sub-equal size with a mean free petal / mean fused petal length ratio = 1.0, petal blotches absent or present, stamens (roughly) the same size as the perianth, 115–186 mm long, style 132–220 mm long, stigma 20-25 mm long, ovoid to ovoid-pointed with a basal constriction. *Infructescences* compact (bract bases imbricate at all stages of maturity), torn and degraded bracts on mature infructescence, old infructescences deciduous, 5–14 fruits per bract. *Fruits* 80–120 $\times$ 32–45 mm, trilocular septifragal capsule, apices conical (Fig. 2b), seeds 6–10 $\times$ 3.2–6 mm, shiny, dark brown, mostly globose, varying in shape according to their distribution in the capsule, ultramarine blue aril.

**Figure 7:**
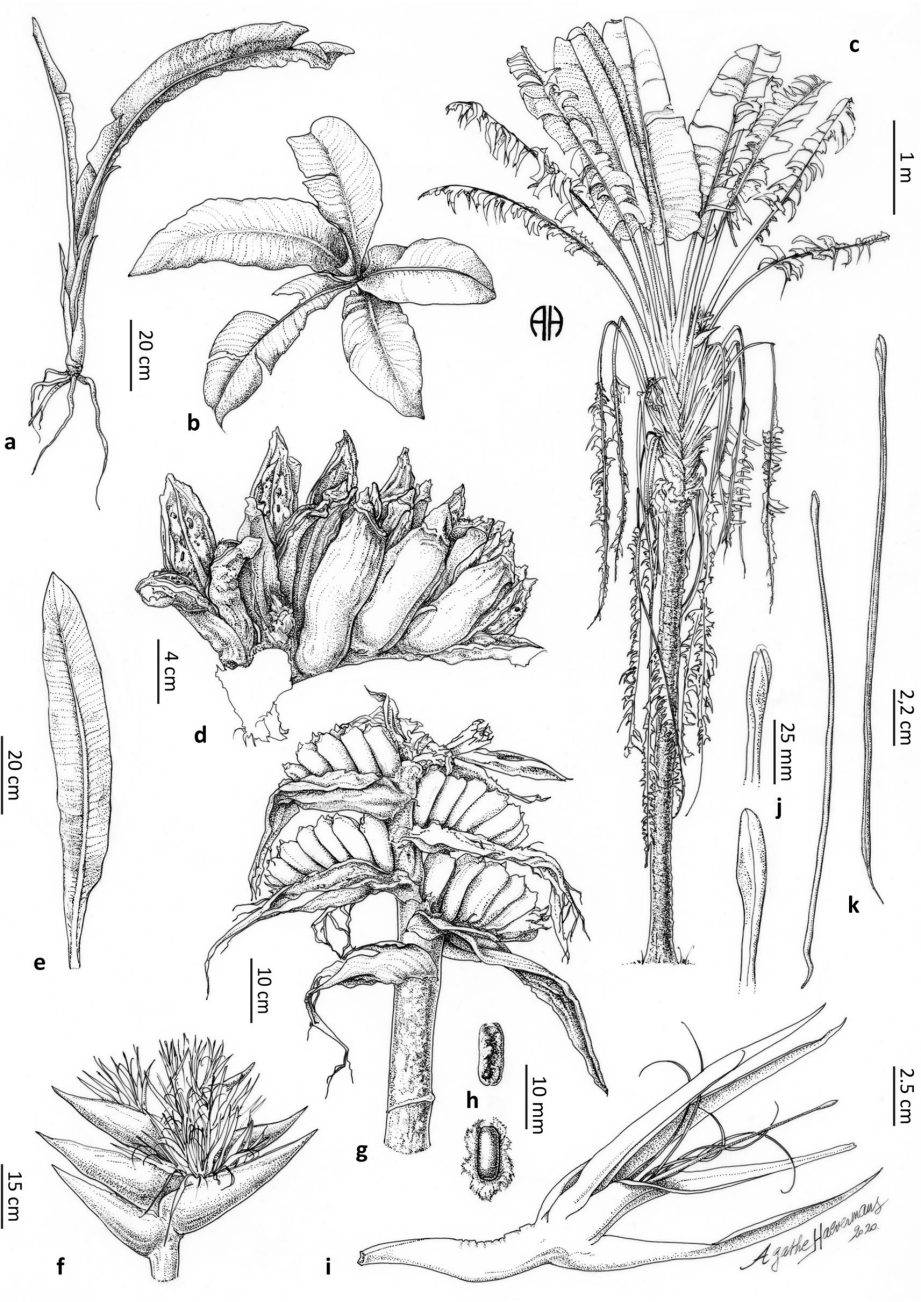
*Ravenala blancii*. (**a**) juvenile plant habit with roots. (**b**) juvenile plant showing the arrangement of laminae. (**c**) adult plant habit. (**d**) mature infructescence segment. (**e**) juvenile leaf showing the attenuate base of the lamina. (**f**) inflorescence with sub-simultaneous opening of the flowers. (**g**) young infructescence with already degraded bracts. (**h**) seeds with arilla. (**i**) open flower. (**j**) details of the stigma. (**k**) style. Ink drawings on $75 \, \upmu$ polyester tracing paper by Agathe Haevermans© from specimens *Hladik 6790, 6239, 6650, Haevermans et al. 832*, and observations in-situ.

**Note** The strong leaf dimorphism between juvenile and adult forms is characteristic of this species^13^, a phenomenon which is not present in the other taxa. The base of the juvenile plant usually grows buried in the leaf litter due to the action of traction roots^13^, its decurrent leaves (Fig. 7) giving it the aspect of a bird’s nest fern.

#### *Ravenala grandis* Haev., Razanats., A. Hladik & P. Blanc *sp. nov.*—Figs. 2c, 3c, 4c, 5c

**Type.** MADAGASCAR $\bullet$ Ampasimbe Commune, Maromaniry Fokontany, along Route Nationale, 18°57′41.8″S, 48°42′41.4″E, 258 m elev., 08 November 2018, *T. Haevermans, A. Haevermans & J. Razanatsoa 831* (Holotype: TAN!, Isotypes: K!, MO!, P!).

**Paratypes** MADAGASCAR $\bullet$ Varifoana, près d’Ambohimahasoa-sud, 15 May 1964, *R. Capuron 26014SF* (P02234597!) $\bullet$ Soanierana-Antasibe[Andasibe], 350 m elev., 10 December 1938, *H.J. Lam & A.D.J. Meeuse 5867* (L-WAG.1111450!, L-WAG.1111451!, L-WAG.1111452!, L-WAG.1111453!, L-L.1477714!, L-L.1477715!).

**Diagnosis** Similar to *Ravenala madagascariensis* but differs in its non-suckering habit, much larger dimensions, very thick leathery laminae, very waxy dark green-yellowish petioles, much larger bracts and overall dimensions, whitish/pure white perianth, strong reddish-pink stripes on its bracteoles, cylindrical stigma without basal constriction, stamens much shorter than perianth, and fruit with a truncated apex.

**Distribution** Eastern rainforests at around 200–500 m elevation in Madagascar^13,20^.

**Preliminary IUCN assessments** We propose a Data Deficient status for *R. grandis*; further fieldwork is required to understand its precise distribution and the status of its populations^33^.

**Ecology** This species seems to favor growing in low discontinuous forests on inselbergs^12^ and thrives in secondary degraded vegetation on the slopes of eastern rain forests.

**Etymology** The name of this species is in reference to its stature and habit, the most robust species of *Ravenala* known.

**Description** Plants solitary (never suckering), 20–30 meters tall (adult), trunk circumference (d.b.h.) 30 cm, juvenile and adult laminae distributed in a perfect fan, 15–30 leaves simultaneously alive on the adult plant, usually 3 leaves between inflorescences. *Leaves* adult petiole 390–440 cm long, dark green/light green-yellowish, very waxy (Fig. 3c), sheath margin moderately developed to undeveloped (0–9 mm), entire on young leaves, splitting and dryish when old, petiole/lamina ratio 1.8–(2.2)–2.6, adult lamina 170–230 $\times$ 94–120 cm, light green, juvenile lamina base non-decurrent. *Inflorescences* 4–6 live lateral inflorescences at a time, 100–120 $\times$ 80–100 cm (peduncle excluded), 10–20 bracts per inflorescence, bracts 440–540 $\times$ 140–170 mm, some waxiness (Fig. 4c), margin color uniformly green, cincinnii of *ca.* 20 flowers per bract, flowering sequentially, bracteoles with a strong reddish-pink stripe. *Flowers* 300 mm long (ovary included), inferior ovary 50–70 mm long, perianth whitish/pure white, sepals narrowly triangular 220–240 $\times$ 10–15 mm, sheathing (fused) petals narrowly triangular 210–220 $\times$ 10–12 mm, free petal acicular 150–170 $\times 3$ mm, slightly smaller than the rest of the perianth with a mean free petal / mean fused petal length ratio = 0.8, petal blotches absent, stamens much shorter than the perianth, 180–200 mm long, style 180–210 mm long, stigma 14–16 mm long, oblong without basal constriction (almost indistinguishable from style). *Infructescences* lax (bract bases not imbricate at some stages of maturity), stiff and coriaceous persisting bracts on mature infructescence, old infructescences deciduous, 5–18 fruits per bract. *Fruits* 100–120 $\times$ 35–40 mm, trilocular septifragal capsule, apices truncate (Fig. 2c), seeds shiny, dark brown, mostly globose, varying in shape according to their distribution in the capsule, ultramarine blue aril.

**Note** The leaves of this species are the most robust and tough of all *Ravenala* species, with a thick leathery texture, making it the material of choice for building roofs^35^.

#### *Ravenala hladikorum* Haev., Razanats., V. Jeannoda & P. Blanc *sp. nov.* — Figs. 2f, 3f, 4f, 5f

**Type** MADAGASCAR $\bullet$ Andasibe; 18°56′00″S, 48°25′06″E; 940 m elev.; 05 February 2004; *A. Hladik & C.-M. Hladik 6842* (Holotype: TAN!, Isotype: P!). **Paratypes.** MADAGASCAR $\bullet$ Andasibe; 18°56′00″S, 48°25′06″E; 940 m elev.; 23 August 1998; *A. Hladik & al. 6240* (fruit with seeds: P!). $\bullet$ Andasibe-Mantadia area, Vakôna, Kalonora; 18°53′17.3″S, 48°25′51.3″E; 934 m elev., 08 November 2018; *T. Haevermans & al. 833* (TAN!, P!, K!, MO!).

**Diagnosis** Similar to *Ravenala madagascariensis* but differs in its non-suckering habit, the alternate positioning of its adult laminae, its dark green leaves, non-waxy petioles with their very papyraceous petiole sheath margins, more than 1 cm long, smaller lamina dimensions, smaller number of simultaneously live inflorescences, purple stripe on bracts and on bracteoles, non-waxy inflorescences, smaller inflorescences, dense infructescences, truncated fruit apices, and short flowering period from November to December.

**Distribution** Andasibe, Mantady, Ranomafana^21^. Restricted to Madagascar.

**Preliminary IUCN assessments** We propose a Data Deficient status for *R. hladikorum*; further fieldwork is required to understand its precise distribution and the status of its populations^33^.

**Ecology** High-elevation species found in eastern rainforests at elevations between 600 and 1100 m. The species seems to favor cool tropical humid and shady conditions.

**Etymology** This species is named in honor of Annette and Claude-Marcel Hladik from the Muséum National d’Histoire Naturelle in Paris, who dedicated their lives to the study of Madagascan biodiversity and contributed greatly to the discovery of this species.

**Description** Plants solitary (never suckering), 10–15 meters tall (adult), trunk circumference (d.b.h.) 20–30 cm, juvenile laminae distributed like a fan, adult laminae arranged in an irregular fan, 9–18 leaves simultaneously alive on the adult plant, 1–3 leaves between inflorescences. *Leaves* adult petiole 280–440 cm long, greenish-yellow, not waxy (Fig. 3f), sheath margin very developed (10 mm and more), split, very papyraceous with min. 1 cm brown dry expansions, petiole/lamina ratio 2.1–(2.42)–2.8, adult lamina 120–160 $\times$ 102–116 cm, dark green, juvenile lamina base non-decurrent. *Inflorescences* 2–3 live lateral inflorescences at a time, $60 \times 90$ cm (peduncle excluded), 4–7 bracts per inflorescence, bracts 150–510 $\times$ 64–100 mm, no waxiness (Fig. 4f), margin green with a purple stripe, cincinnii of 5–14 flowers per bract, sequentially flowering, bracteoles with a dark purple colored stripe. *Flowers* 240–320 mm long (ovary included), inferior ovary 40–60 mm long, perianth whitish, sepals narrowly triangular 210–265$\times$*ca.* 10 mm, sheathing (fused) petals narrowly triangular 190–240$\times$*ca.* 10 mm , free petal acicular 135–220 $\times$ 5 mm, almost the same size as the fused petals with a mean free petal / mean fused petal length ratio = 0.9, petal blotches unknown, stamens (roughly) the same size as the perianth, 170–230 mm long, style 187–250 mm long, stigma 20–25 mm long, ovoid with a basal constriction. *Infructescences* compact (bract bases imbricate at all stages of maturity), stiff and coriaceous persistent bracts on mature infructescences, old infructescences deciduous, 5–14 fruits per bract. *Fruits* 82–108 $\times$ 34–48 mm, trilocular septifragal capsule, apices truncate (Fig. 2f), seeds 4–9 $\times$ 3–6 mm, shiny, dark brown, mostly globose, varying in shape according to their distribution in the capsule, ultramarine blue aril.

#### *Ravenala menahirana* Haev. & Razanats. *sp. nov.*—Figs. 2e, 3e, 4e, 5e

**Type** MADAGASCAR $\bullet$ Foulpointe, Analalava Forest; 17°42.3′S, 49°27.38′E; 50 m elev.; 20 March 2016; *T.Haevermans, M. Vorontsova, S. Dransfield & J. Razanatsoa 826* (Holotype: TAN!, Isotypes: P!, K !, MO!).

**Diagnosis** Similar to *Ravenala madagascariensis* but differs in its non-suckering habit, the alternate dark green laminae tending not to form a perfect fan (Fig. 5e), dark red petioles with a zigzagging well developed dryish sheath margin, more strongly obovoid laminae, smaller number of simultaneously live inflorescences, smaller inflorescences tinged with red, pure white/whitish perianth, smaller flowers, dense infructescences, the fruit apices truncate with a mucro, and subequal free and fused petals.

**Distribution** Appears to be restricted to the east coast in the area around Analalava-Foulpointe up to the Mananara-Avaratra area. Two human observations from Marojejy (North-East) and Tampolo (Masoala) seem also to be this species. Restricted to Madagascar.

**Preliminary IUCN assessments** We propose a Data Deficient status for *R. menahirana*; further fieldwork is required to understand its precise distribution and the status of its populations^33^.

**Ecology** This coastal forest-dwelling species favors low-elevation tropical humid conditions in the Analalava-Foulpointe area, extending north to Mananara-Avaratra area, and maybe up to Marojejy.

**Etymology** The name of this species is in reference to one of its local names “*menahirana*”, given to the species in the Analalava-Foulpointe area and meaning “red ravenala”.

**Description** Plants solitary (never suckering), 6–10 meters tall (adult), trunk circumference (d.b.h.) 20–30 cm, juvenile laminae distributed like a fan, adult laminae arranged in an irregular to regular fan, 12–18 leaves simultaneously alive on the adult plant, 3 leaves between inflorescences. *Leaves* adult petiole 200–230 cm long, dark red, slightly to very waxy, sheath margin very developed (10 mm and more), red, entire, forming a three dimensional zigzag pattern (Fig. 3e), then splitting and drying on old leaves, petiole/lamina ratio 1.4–(1.7)–1.9, adult lamina $350 \times 120$ cm, lamina color dark green, juvenile lamina base non-decurrent. *Inflorescences* 1–2 live lateral inflorescences at a time, $60 \times 70$ cm (peduncle excluded), 10–12 bracts per inflorescence, bracts 260–360 $\times$ 50–80 mm, very waxy (Fig. 4e), margin color uniformly reddish-green, cincinnii of 8–12 flowers per bract, flowering sequentially, no colored stripe on bracteoles (apices sometimes suffused with pink). *Flowers* 220–250 mm long (ovary included), inferior ovary 40–60 mm long, perianth pure white to whitish, sepals narrowly triangular 180–230 $\times$ 12–16 mm, sheathing (fused) petals narrowly triangular 160–180 $\times$ 5 mm, free petal acicular 160–170 $\times$ 5 mm, free petal the same size as the remaining perianth with a mean free petal / mean fused petal length ratio = 1.0, petal blotches absent, stamens the same size (roughly) as the perianth, stamen 150–160 mm long, style 150–200 mm long, stigma 10 mm long, oblong with a basal constriction. *Infructescences* compact (bract bases imbricate at all stages of maturity), stiff and coriaceous persisting bracts on mature infructescences, old infructescences deciduous, 8–12 fruits per bract. *Fruits* 80–100 $\times$ 30–35 mm, trilocular septifragal capsule, apices truncate with a mucro (Fig. 2e), seeds shiny, dark brown, mostly globose, varying in shape according to their distribution in the capsule, ultramarine blue aril.

**Note** This species is similar to *R. hladikorum* but is easily distinguished by, in addition to its petioles and its ecology, its truncate mucronate fruit apices, the shape of the synflorescence bracts and the absence of a red stripe on the cyme bracteoles.

### Identification key to the species of genus *Ravenala*


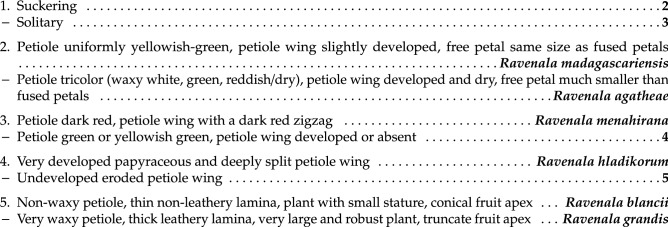


## Discussion

The five new species described in this paper can be easily identified at both young and adult stages from distinctive morphological characters defined from petiole sheath margins, petiole coloring and morphology, suckering or solitary habits and flower and fruit traits. The main stable character distinguishing these species^13^ is the ability to produce suckers (*R. agatheae*, *R. madagascariensis*) like other members of Zingiberales, or its absence (i.e. strictly monopodial) (*R. blancii*, *R. grandis*, *R. hladikorum*, *R. menahirana*) (Table 1). The laminae arrangement in space is also of taxonomic significance, especially for the juvenile *R. blancii*, whose leaves, although distichally inserted, have laminae distributed in such a way as to form a torus (Fig. 7b), which is likely to be an adaptation to the low light intensity of the forest floor where it germinates^12^. At early stages, this species, with its decurrent laminae arranged in a torus, bears a striking resemblance to the bird’s nest fern (*Asplenium nidus* Linnaeus^36^). The genus occurs across a variety of bioclimates and habitats, with each species seemingly favoring a specific niche. Both suckering species occur at sea level and on low-elevation coastal hills, with the well-known *R. madagascariensis* being documented in the coastal marshes of the east coast of the island, whereas *R. agatheae* is documented in the north-western part of the island (Fig. 1). The comparatively drier climates in which the latter occurs may favor the persistence on the “trunk” of a blanket of dry petioles and infructescences which may be involved in some form of fire resistance. By contrast, the solitary species are all distributed on the eastern part of Madagascar from sea level to around 1000 m elevation, on slopes originally covered by forests. Their distribution likely follows an elevation gradient, with *R. menahirana* found in now rare sea level coastal forests, *R. grandis* in mid-elevation (300–500 m) forests on steep inselbergs slopes (see Figs. 2, 3, and 4 in Blanc et al.^12^), while *R. blancii* and *R. hladikorum* are found in sympatry in the shade in high elevation (1000 m) rain forests^12^. Further research on the population genetics, pollination and seed dispersal of the formerly monotypic *Ravenala* would be essential within this new six-species framework. This genus may also prove to be a good model for studying sympatric speciation and provide a better understanding of the interactions of the endemic fauna of Madagascar with this flagship taxon.

**Table 1:** Comparison of the main distinctive morphological characters for the six species of *Ravenala*.

	*R. agatheae*	*R. blancii*	*R. grandis*	*R. hladikorum*	*R. madagascariensis*	*R. menahirana*
Habit	Suckering	Solitary	Solitary	Solitary	Suckering	Solitary
Adult dimensions (m)	6–10	10–15	20–30	10–15	6–12	6–10
Leaves lamina color	Dark green	Dark green	Light green	Dark green	Light green	Dark green
Leaves simultaneously alive	9–22	9–16	15–30	9–18	14–26	12–18
Petiole color	Tricolor (white, green, red)	Green	Dark green/Yellowish	Greenish-yellow	Yellowish green	Dark red
Sheath margin dvp.	Very developed (10 mm+)	Undeveloped	Developped (0–9 mm)	Very developed (10 mm+)	Developed (0–9 mm)	Very developed (10 mm+)
Sheath margin shape	Dryish papyraceous	Eroded	Entire/split	Brown papyraceous, split	Not dry, entire/split	Dark red dry zigzag
Petiole & dry infr. persistence	Yes	No	No	No	No	No
Juvenile lamina	Non-decurrent	Decurrent	Non-decurrent	Non-decurrent	Non-decurrent	Non-decurrent
Juvenile lamina distribution	Irregular fan	Torus shape	Irregular fan	Irregular fan	Irregular fan	Irregular fan
Adult leaves distribution	Regular fan	Regular fan	Regular fan	Irregular fan	Regular fan	Irregular fan
Bracts #/Infl.	10–14	4–6	10–20	4–7	8–16	10–12
Bracts L/l	450–500 $\times$ 80–90 (5.3)	160–350 $\times$ 50–90 (3.9)	440–540 $\times$ 140–170 (3.16)	150–510 $\times$ 140–170 (2.3)	200–450 $\times$ 50–100 (4.3)	260–360 $\times$ 50–80 (4.8)
Bracts persistence	Siff and coriaceous	Torn and degraded	Siff and coriaceous	Siff and coriaceous	Siff and coriaceous	Siff and coriaceous
Infructescence imbrication	Compact	Compact	Lax	Compact	Lax	Compact
Flower length (+ovary) in mm	260–310	165–280	300	240–320	240–280	220–250
Free vs fused petals ratio	Much shorter (0.6)	Subequal (1)	Slightly smaller (0.8)	Slightly smaller (0.9)	Slightly smaller (0.8)	Subequal (1)
Stamens size vs perianth	Similar	Similar	Much shorter	Similar	Similar	Similar
Fruit apex	Conical	Conical	Truncated	Truncated	Conical	Truncated with bony mucro

## Methods

Taxonomic treatment follows the International Code of Nomenclature (ICN) for algae, fungi and plants^37^ (Shenzhen Code). Specimen citations follow the CETAF (Consortium of European Taxonomic Facilities) guidelines^38^. Specimens were deposited in natural history collections as indicated by their international acronym^39^. Descriptive terms follow standard botanical terminology^40^. Macromorphological characters (see a summary in Table 1) were studied in the field and in the laboratory from specimens the authors collected, according to national and international standards and regulation, and from specimens held in several herbaria where *Ravenala* specimens are deposited (G, K, MO, P, TAN, US)^39^. The distribution maps (Fig. 1) were prepared with the software R, using coordinates from our own collections and observation data, from other available specimens (*n* = 19 gatherings, represented as *n* = 40 sheets), as well as from the www.inaturalist.org website observations we managed to identify (*n* = 83). We used the triple equality sign ($\equiv$) to indicate homotypic synonyms, the equal sign (=) to indicate heterotypic synonyms and the ‘en-dash’ (–) to indicate invalid names. All protologues of relevant names were consulted to establish the nomenclatural synopsis.
